# 

**Published:** 1885-09-20

**Authors:** 


Particularly indicated in Cholera Infantum. Diarrhœa and Stomach Disorders

THE CHAS H PHILLIPS CHEMICAL CO., 30 Platt St., N. Y.

VOL. XV. SEPTEMBER 20, 1885. NO. 9

LIBRARY
INDEXED
S.G.O.

### THE SOUTHERN MEDICAL RECORD:

### A MONTHLY JOURNAL OF PRACTICAL MEDICINE.

Terms: $2.00 per Annum, in Advance; 4 Copies $6.00; Single Copies 20 Cents.

INDEX
MEDICUS

*"QUICQUID PRÆCIPIES ESTO BREVIS."*

Address R. C. WORD, M.D., Managing Editor, Atlanta, Ga.

Entered at the Post-Office at Atlanta, as second-class matter.

